# Ethyl 3-oxo-3*H*-benzo[*f*]chromene-2-carboxyl­ate

**DOI:** 10.1107/S1600536810037840

**Published:** 2010-09-30

**Authors:** M. Vindu Vahini, H. C. Devarajegowda, K. M. Mahadevan, T. G. Meenakshi, H. K. Arunkashi

**Affiliations:** a Department of Physics, Sri D Devaraja Urs Govt. First Grade College, Hunsur 571 105, Mysore District, Karnataka, India; b Department of Physics, Yuvaraja’s College (Constituent College), University of Mysore, Mysore 570 005, Karnataka, India; c Department of PG Studies in Pharmaceutical Chemistry, Kuvempu University Kadur P. G. Center, Kadur 577 548, Karnataka, India; d Department of Physics, Y. Y. D. Govt. First Grade College, Belur 573 115, Hassan, Karnataka, India

## Abstract

In the title compound, C_16_H_12_O_4_, the chromene ring system is almost planar [maximum deviation = 0.026 (1) Å] and makes dihedral angles of 1.24 (9) and 26.5 (2)° with the fused benzene ring and the plane of the ethyl carboxyl­ate group, respectively.

## Related literature

For general background to chromenes, see: Kendall *et al.* (1961 ▶); Rau & Brack (1963 ▶); Jones *et al.* (1985 ▶); Gikas *et al.* (2003 ▶); Miyata & Nalwa, (1997 ▶); Shibata (1994 ▶). For related structures, see: Lakshmi *et al.* (2006 ▶); Jiao *et al.* (2009 ▶).
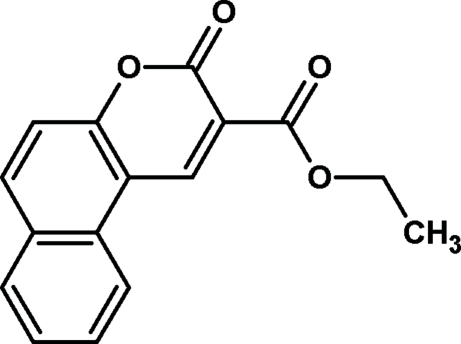


## Experimental

### 

#### Crystal data

C_16_H_12_O_4_
*M* *_r_* = 268.26
Monoclinic, 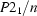

*a* = 14.6716 (11) Å
*b* = 4.5190 (5) Å
*c* = 19.3874 (18) Å
β = 90.218 (7)°
*V* = 1285.4 (2) Å^3^
*Z* = 4
Mo *K*α radiation
μ = 0.10 mm^−1^
*T* = 293 K
0.22 × 0.15 × 0.12 mm

#### Data collection

Bruker SMART CCD area-detector diffractometer
Absorption correction: ψ scan (*SADABS*; Sheldrick, 2004 ▶) *T*_min_ = 0.981, *T*_max_ = 0.987
13075 measured reflections
2276 independent reflections
997 reflections with *I* > 2σ(*I*)
*R*_int_ = 0.088

#### Refinement

*R*[*F* ^2^ > 2σ(*F* ^2^)] = 0.075
*wR*(*F* ^2^) = 0.208
*S* = 1.04
2276 reflections
182 parameters
H-atom parameters constrained
Δρ_max_ = 0.17 e Å^−3^
Δρ_min_ = −0.16 e Å^−3^

### 

Data collection: *SMART* (Bruker, 2001 ▶); cell refinement: *SAINT* (Bruker, 2001 ▶); data reduction: *SAINT*; program(s) used to solve structure: *SHELXS97* (Sheldrick, 2008 ▶); program(s) used to refine structure: *SHELXL97* (Sheldrick, 2008 ▶); molecular graphics: *ORTEP-3* (Farrugia, 1999 ▶); software used to prepare material for publication: *SHELXL97*.

## Supplementary Material

Crystal structure: contains datablocks I, global. DOI: 10.1107/S1600536810037840/wn2410sup1.cif

Structure factors: contains datablocks I. DOI: 10.1107/S1600536810037840/wn2410Isup2.hkl

Additional supplementary materials:  crystallographic information; 3D view; checkCIF report

## Figures and Tables

**Fig. 1.**
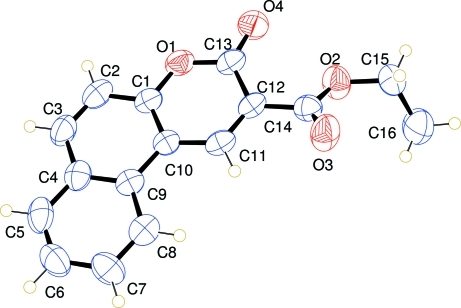
The asymmetric unit of the title compound. Displacement ellipsoids are drawn at the 50% probability level. Hydrogen atoms are shown as spheres of arbitrary radius.

**Fig. 2.**
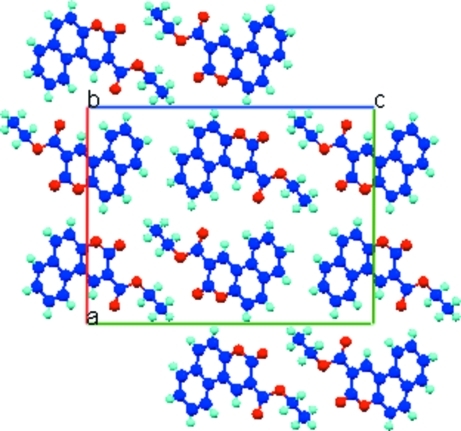
Packing of the molecules when viewed along b.

## supplementary crystallographic information

### Comment

Chromenes are involved in the structures of molecules of biologically active compounds. Several chromene derivatives have been patented for a variety of industrial applications (Kendall *et al.*, 1961; Rau & Brack,1963).

Chromenes also play a vital role in electrophotographic, electroluminescent devices and laser dyes. Several 3-substituted 7-hydroxycoumarins rank among the most efficient photostable laser dyes, emitting in the blue green region of the visible spectrum. The lasing range covered by chromene dyes is appreciably extended when the 3-substituent is a heterocyclic unit (Jones *et al.*, 1985; Gikas *et al.*, 2003). Benzo-annulated chromene derivativesare widely used in organic light-emitting devices and are used as electron-transporting emitters (Miyata & Nalwa *et al.*, 1997; Shibata, 1994).

In the title compound (Fig. 1) the chromene ring system is almost planar [maximum deviation = 0.026 (1) Å] and makes dihedral angles of 1.24 (9)° and 26.5 (2)% with the fused benzene ring and the plane of the ethyl carboxylate group, respectively. The bond lengths and bond angles are in good agreement with those in related structures (Lakshmi *et al.*, 2006; Jiao *et al.*, 2009). The packing of the molecules, when viewed along *b*, is shown in Fig. 2.

### Experimental

A mixture of 2-hydroxy-1-naphthaldehyde (2.9 mmol), an equivalent amount of diethyl malonate (2.9 mmol), and a catalytic amount of piperidine in ethanol (30 ml) was refluxed for 30 minutes on a water bath. After the reaction was complete, the reaction mixture was cooled to room temperature and poured into crushed ice with stirring. The precipitate obtained was then filtered, washed with water, dried and recrystallized using ethanol to yield the pure title compound as a white-yellow crystalline solid. Yield 90%; m.p. 388 K.

### Refinement

All H atoms were positioned geometrically with C—H = 0.93 Å for aromatic H, 0.97 Å for methylene H and 0.96 Å for methyl H and refined using a riding model with *U*_iso_(H) =1.5*U*_eq_(C) for methyl H and 1.2*U*_eq_(C) for all other H.

### Crystal data

**Table:**

C_16_H_12_O_4_	*F*(000) = 560
*M**_r_* = 268.26	*D*_x_ = 1.386 Mg m^−^^3^
Monoclinic, *P*2_1_/*n*	Melting point < 388 K
Hall symbol: -P 2yn	Mo *K*α radiation, λ = 0.71073 Å
*a* = 14.6716 (11) Å	Cell parameters from 2276 reflections
*b* = 4.5190 (5) Å	θ = 2.8–25.0°
*c* = 19.3874 (18) Å	µ = 0.10 mm^−^^1^
β = 90.218 (7)°	*T* = 293 K
*V* = 1285.4 (2) Å^3^	Plate, colourless
*Z* = 4	0.22 × 0.15 × 0.12 mm

### Data collection

**Table:**

Bruker SMART CCD area-detector diffractometer	2276 independent reflections
Radiation source: fine-focus sealed tube	997 reflections with *I* > 2σ(*I*)
graphite	*R*_int_ = 0.088
ω and φ scans	θ_max_ = 25.0°, θ_min_ = 2.8°
Absorption correction: ψ scan (*SADABS*; Sheldrick, 2004)	*h* = −17→17
*T*_min_ = 0.981, *T*_max_ = 0.987	*k* = −5→5
13075 measured reflections	*l* = −23→23

### Refinement

**Table:**

Refinement on *F*^2^	Secondary atom site location: difference Fourier map
Least-squares matrix: full	Hydrogen site location: inferred from neighbouring sites
*R*[*F*^2^ > 2σ(*F*^2^)] = 0.075	H-atom parameters constrained
*wR*(*F*^2^) = 0.208	*w* = 1/[σ^2^(*F*_o_^2^) + (0.0891*P*)^2^] where *P* = (*F*_o_^2^ + 2*F*_c_^2^)/3
*S* = 1.03	(Δ/σ)_max_ < 0.001
2276 reflections	Δρ_max_ = 0.17 e Å^−^^3^
182 parameters	Δρ_min_ = −0.16 e Å^−^^3^
0 restraints	Extinction correction: *SHELXL*, Fc^*^=kFc[1+0.001xFc^2^λ^3^/sin(2θ)]^-1/4^
Primary atom site location: structure-invariant direct methods	Extinction coefficient: 0.017 (4)

### Special details

**Table:**

Experimental. ^1^H NMR (400 MHz, CDCl_3_) δ(p.p.m.): 1.4 (s, 3H, CH_3_), 4.4 (q, 2H, H2), 7.4 (m, 6H, ArH), 9.2 (s, 1H, CH): ^13^C NMR (300 MHz, CDCl_3_) δ(p.p.m.): 163 (C=O ester), 160 (C=O pyrone), 150.4, 149.7, 148.7, 130.6, 126.6, 124.2, 124.0, 121.2, 121, 120, 114.5, 115, 58.8, 26: IR (KBr) ν(cm^-1^): 1765 (*s*) (C=O), 1750 (*s*) (C=O ester): MS (m/*z*): 269 (*M*+1).
Geometry. All s.u.'s (except the s.u. in the dihedral angle between two l.s. planes) are estimated using the full covariance matrix. The cell s.u.'s are taken into account individually in the estimation of s.u.'s in distances, angles and torsion angles; correlations between s.u.'s in cell parameters are only used when they are defined by crystal symmetry. An approximate (isotropic) treatment of cell s.u.'s is used for estimating s.u.'s involving l.s. planes.
Refinement. Refinement of *F*^2^ against ALL reflections. The weighted *R*-factor *wR* and goodness of fit *S* are based on *F*^2^, conventional *R*-factors *R* are based on *F*, with *F* set to zero for negative *F*^2^. The threshold expression of *F*^2^ > 2σ(*F*^2^) is used only for calculating *R*-factors(gt) *etc*. and is not relevant to the choice of reflections for refinement. *R*-factors based on *F*^2^ are statistically about twice as large as those based on *F*, and *R*- factors based on ALL data will be even larger.

### Fractional atomic coordinates and isotropic or equivalent isotropic displacement parameters (Å^2^)

**Table:**

	*x*	*y*	*z*	*U*_iso_*/*U*_eq_	
O1	0.37862 (18)	−0.4741 (7)	−0.03118 (17)	0.0587 (9)	
O2	0.19329 (19)	−0.8301 (8)	−0.17429 (17)	0.0700 (10)	
O3	0.0829 (2)	−0.7805 (8)	−0.09664 (17)	0.0802 (12)	
O4	0.3625 (2)	−0.8050 (8)	−0.11371 (17)	0.0777 (11)	
C1	0.3450 (3)	−0.2819 (10)	0.0168 (2)	0.0518 (12)	
C2	0.4098 (3)	−0.1524 (12)	0.0605 (3)	0.0657 (14)	
H2	0.4714	−0.1975	0.0560	0.079*	
C3	0.3813 (3)	0.0397 (11)	0.1094 (3)	0.0641 (14)	
H3	0.4241	0.1276	0.1383	0.077*	
C4	0.2879 (3)	0.1105 (10)	0.1176 (2)	0.0535 (13)	
C5	0.2597 (4)	0.3160 (11)	0.1686 (2)	0.0671 (15)	
H5	0.3025	0.4041	0.1975	0.081*	
C6	0.1708 (4)	0.3826 (12)	0.1749 (3)	0.0731 (15)	
H6	0.1527	0.5166	0.2086	0.088*	
C7	0.1050 (4)	0.2543 (12)	0.1319 (3)	0.0765 (16)	
H7	0.0439	0.3042	0.1367	0.092*	
C8	0.1309 (3)	0.0572 (11)	0.0833 (3)	0.0634 (14)	
H8	0.0868	−0.0285	0.0551	0.076*	
C9	0.2229 (3)	−0.0214 (9)	0.0743 (2)	0.0467 (11)	
C10	0.2530 (3)	−0.2264 (9)	0.0221 (2)	0.0444 (11)	
C11	0.1960 (3)	−0.3847 (10)	−0.0243 (2)	0.0503 (12)	
H11	0.1334	−0.3554	−0.0213	0.060*	
C12	0.2266 (3)	−0.5729 (10)	−0.0719 (2)	0.0497 (12)	
C13	0.3241 (3)	−0.6306 (12)	−0.0766 (2)	0.0545 (13)	
C14	0.1601 (3)	−0.7379 (9)	−0.1150 (2)	0.0533 (12)	
C15	0.1339 (3)	−1.0111 (12)	−0.2181 (3)	0.0715 (15)	
H15A	0.0993	−1.1455	−0.1892	0.086*	
H15B	0.1711	−1.1291	−0.2488	0.086*	
C16	0.0699 (4)	−0.8298 (13)	−0.2596 (3)	0.0918 (18)	
H16A	0.0327	−0.9573	−0.2876	0.138*	
H16B	0.1038	−0.6984	−0.2887	0.138*	
H16C	0.0317	−0.7164	−0.2294	0.138*	

### Atomic displacement parameters (Å^2^)

**Table:**

	*U*^11^	*U*^22^	*U*^33^	*U*^12^	*U*^13^	*U*^23^
O1	0.0418 (18)	0.068 (2)	0.066 (2)	0.0077 (16)	0.0000 (17)	−0.0002 (18)
O2	0.062 (2)	0.089 (3)	0.059 (2)	−0.0067 (18)	0.0089 (18)	−0.017 (2)
O3	0.046 (2)	0.119 (3)	0.075 (2)	−0.0087 (19)	0.0054 (18)	−0.028 (2)
O4	0.064 (2)	0.097 (3)	0.072 (2)	0.0201 (19)	0.0008 (19)	−0.021 (2)
C1	0.045 (3)	0.057 (3)	0.054 (3)	0.008 (2)	−0.002 (2)	0.005 (3)
C2	0.043 (3)	0.078 (4)	0.076 (4)	0.001 (3)	−0.010 (3)	0.009 (3)
C3	0.054 (3)	0.065 (4)	0.073 (4)	−0.009 (3)	−0.020 (3)	0.000 (3)
C4	0.064 (3)	0.054 (3)	0.043 (3)	−0.005 (2)	−0.005 (2)	0.013 (2)
C5	0.091 (4)	0.064 (4)	0.046 (3)	−0.011 (3)	−0.017 (3)	0.008 (3)
C6	0.092 (4)	0.072 (4)	0.056 (3)	0.005 (3)	0.008 (3)	−0.015 (3)
C7	0.082 (4)	0.089 (4)	0.059 (3)	0.010 (3)	0.020 (3)	−0.016 (3)
C8	0.058 (3)	0.067 (4)	0.065 (3)	−0.002 (3)	−0.004 (3)	0.006 (3)
C9	0.045 (3)	0.048 (3)	0.047 (3)	−0.001 (2)	0.003 (2)	0.012 (2)
C10	0.044 (3)	0.043 (3)	0.045 (3)	−0.001 (2)	0.001 (2)	0.009 (2)
C11	0.042 (3)	0.059 (3)	0.050 (3)	0.007 (2)	0.003 (2)	0.015 (3)
C12	0.043 (3)	0.057 (3)	0.049 (3)	0.005 (2)	−0.004 (2)	0.012 (3)
C13	0.049 (3)	0.065 (4)	0.050 (3)	0.009 (3)	−0.002 (3)	0.011 (3)
C14	0.055 (3)	0.052 (3)	0.052 (3)	0.005 (3)	0.001 (3)	0.004 (2)
C15	0.074 (3)	0.073 (4)	0.068 (3)	0.004 (3)	−0.008 (3)	−0.020 (3)
C16	0.110 (5)	0.090 (5)	0.075 (4)	0.006 (3)	−0.025 (3)	−0.005 (3)

### Geometric parameters (Å, °)

**Table:**

O1—C1	1.366 (5)	C6—H6	0.9300
O1—C13	1.381 (5)	C7—C8	1.353 (6)
O2—C14	1.318 (5)	C7—H7	0.9300
O2—C15	1.464 (5)	C8—C9	1.408 (6)
O3—C14	1.204 (5)	C8—H8	0.9300
O4—C13	1.208 (5)	C9—C10	1.443 (6)
C1—C10	1.376 (5)	C10—C11	1.420 (6)
C1—C2	1.400 (6)	C11—C12	1.333 (6)
C2—C3	1.352 (6)	C11—H11	0.9300
C2—H2	0.9300	C12—C13	1.458 (6)
C3—C4	1.417 (6)	C12—C14	1.483 (6)
C3—H3	0.9300	C15—C16	1.481 (6)
C4—C9	1.400 (6)	C15—H15A	0.9700
C4—C5	1.419 (6)	C15—H15B	0.9700
C5—C6	1.345 (7)	C16—H16A	0.9600
C5—H5	0.9300	C16—H16B	0.9600
C6—C7	1.399 (7)	C16—H16C	0.9600
			
C1—O1—C13	123.3 (3)	C8—C9—C10	123.0 (4)
C14—O2—C15	117.5 (4)	C1—C10—C11	115.9 (4)
O1—C1—C10	121.5 (4)	C1—C10—C9	118.1 (4)
O1—C1—C2	115.6 (4)	C11—C10—C9	125.9 (4)
C10—C1—C2	122.9 (5)	C12—C11—C10	124.0 (4)
C3—C2—C1	118.7 (5)	C12—C11—H11	118.0
C3—C2—H2	120.6	C10—C11—H11	118.0
C1—C2—H2	120.6	C11—C12—C13	119.4 (4)
C2—C3—C4	121.7 (5)	C11—C12—C14	119.2 (4)
C2—C3—H3	119.1	C13—C12—C14	121.2 (5)
C4—C3—H3	119.1	O4—C13—O1	116.3 (4)
C9—C4—C3	119.5 (5)	O4—C13—C12	127.9 (5)
C9—C4—C5	119.8 (4)	O1—C13—C12	115.8 (4)
C3—C4—C5	120.7 (5)	O3—C14—O2	123.9 (4)
C6—C5—C4	119.7 (5)	O3—C14—C12	122.1 (4)
C6—C5—H5	120.2	O2—C14—C12	114.0 (4)
C4—C5—H5	120.2	O2—C15—C16	112.4 (4)
C5—C6—C7	121.3 (5)	O2—C15—H15A	109.1
C5—C6—H6	119.3	C16—C15—H15A	109.1
C7—C6—H6	119.3	O2—C15—H15B	109.1
C8—C7—C6	119.6 (5)	C16—C15—H15B	109.1
C8—C7—H7	120.2	H15A—C15—H15B	107.9
C6—C7—H7	120.2	C15—C16—H16A	109.5
C7—C8—C9	121.6 (5)	C15—C16—H16B	109.5
C7—C8—H8	119.2	H16A—C16—H16B	109.5
C9—C8—H8	119.2	C15—C16—H16C	109.5
C4—C9—C8	118.0 (4)	H16A—C16—H16C	109.5
C4—C9—C10	119.0 (4)	H16B—C16—H16C	109.5
			
C13—O1—C1—C10	−1.5 (6)	C4—C9—C10—C1	−0.5 (6)
C13—O1—C1—C2	177.3 (4)	C8—C9—C10—C1	178.5 (4)
O1—C1—C2—C3	−179.9 (4)	C4—C9—C10—C11	178.0 (4)
C10—C1—C2—C3	−1.1 (7)	C8—C9—C10—C11	−3.1 (6)
C1—C2—C3—C4	0.5 (7)	C1—C10—C11—C12	−1.4 (6)
C2—C3—C4—C9	0.1 (7)	C9—C10—C11—C12	−179.9 (4)
C2—C3—C4—C5	−179.1 (5)	C10—C11—C12—C13	1.8 (6)
C9—C4—C5—C6	0.0 (7)	C10—C11—C12—C14	176.8 (4)
C3—C4—C5—C6	179.2 (4)	C1—O1—C13—O4	−176.1 (4)
C4—C5—C6—C7	−0.3 (7)	C1—O1—C13—C12	1.7 (6)
C5—C6—C7—C8	0.6 (8)	C11—C12—C13—O4	175.6 (5)
C6—C7—C8—C9	−0.5 (7)	C14—C12—C13—O4	0.8 (7)
C3—C4—C9—C8	−179.1 (4)	C11—C12—C13—O1	−1.8 (6)
C5—C4—C9—C8	0.1 (6)	C14—C12—C13—O1	−176.7 (4)
C3—C4—C9—C10	−0.1 (6)	C15—O2—C14—O3	−4.1 (7)
C5—C4—C9—C10	179.1 (4)	C15—O2—C14—C12	176.2 (4)
C7—C8—C9—C4	0.2 (7)	C11—C12—C14—O3	−22.9 (6)
C7—C8—C9—C10	−178.8 (4)	C13—C12—C14—O3	151.9 (4)
O1—C1—C10—C11	1.2 (6)	C11—C12—C14—O2	156.8 (4)
C2—C1—C10—C11	−177.5 (4)	C13—C12—C14—O2	−28.4 (6)
O1—C1—C10—C9	179.8 (4)	C14—O2—C15—C16	82.2 (5)
C2—C1—C10—C9	1.1 (6)		
